# Bisphenol S Promotes the Transfer of Antibiotic Resistance Genes via Transformation

**DOI:** 10.3390/ijms25189819

**Published:** 2024-09-11

**Authors:** Jiayi Zhang, Shuyao Zhu, Jingyi Sun, Yuan Liu

**Affiliations:** 1 Jiangsu Co-Innovation Center for Prevention and Control of Important Animal Infectious Diseases and Zoonoses, College of Veterinary Medicine, Yangzhou University, Yangzhou 225009, China; 2 Joint International Research Laboratory of Agriculture and Agri-Product Safety, the Ministry of Education of China, Yangzhou University, Yangzhou 225009, China; 3 Institute of Comparative Medicine, Yangzhou University, Yangzhou 225009, China

**Keywords:** transformation, bisphenol, antibiotic resistance, ARGs

## Abstract

The antibiotic resistance crisis has seriously jeopardized public health and human safety. As one of the ways of horizontal transfer, transformation enables bacteria to acquire exogenous genes naturally. Bisphenol compounds are now widely used in plastics, food, and beverage packaging, and have become a new environmental pollutant. However, their potential relationship with the spread of antibiotic resistance genes (ARGs) in the environment remains largely unexplored. In this study, we aimed to assess whether the ubiquitous bisphenol S (BPS) could promote the transformation of plasmid-borne ARGs. Using plasmid pUC19 carrying the ampicillin resistance gene as an extracellular ARG and model microorganism *E. coli* DH5α as the recipient, we established a transformation system. Transformation assays revealed that environmentally relevant concentrations of BPS (0.1–10 μg/mL) markedly enhanced the transformation frequency of plasmid-borne ARGs into *E. coli* DH5α up to 2.02-fold. Fluorescent probes and transcript-level analyses suggest that BPS stimulated increased reactive oxygen species (ROS) production, activated the SOS response, induced membrane damage, and increased membrane fluidity, which weakened the barrier for plasmid transfer, allowing foreign DNA to be more easily absorbed. Moreover, BPS stimulates ATP supply by activating the tricarboxylic acid (TCA) cycle, which promotes flagellar motility and expands the search for foreign DNA. Overall, these findings provide important insight into the role of bisphenol compounds in facilitating the horizontal spread of ARGs and emphasize the need to monitor the residues of these environmental contaminants.

## 1. Introduction

Antibiotics are indispensable drugs in modern medicine for the treatment of various bacterial infections. However, with the widespread use of antibiotics, bacterial resistance to antibiotics is gradually becoming a major public health problem worldwide [1]. Globally, today 700,000 people die each year from antibiotic resistance, a number which is expected to expand to one million by 2050. Unlike COVID-19, which broke out with great fanfare, antibiotic resistance is more of a silent pandemic [2].

It is well known that, in addition to the emergence of antibiotic resistance due to mutations under antibiotic stress [3], horizontal gene transfer (HGT) greatly contributes to the spread of antibiotic resistance. There are four recognized methods of HGT, i.e., conjugation, transformation, transduction, and vesiduction, which contribute to the sharing of antibiotic resistance genes (ARGs) between bacteria. The remarkable aspect of the transformation is that no direct contact between bacteria or even a living donor is required; thus, an environment abundant in free DNA serves as the optimal incubator. It is often assumed that sub-inhibitory concentrations of antibiotics are the major driver of antibiotic resistance transmission [4]. However, recent studies have shown that in addition to antibiotic factors, the roles of non-antibiotic factors in the spread of resistance should not be underestimated, e.g., disinfectants, triclosan, carbon dioxide, and artificial sweeteners [5,6,7,8].

With the rapid development of recent industries, the bisphenol compound bisphenol A (BPA) is ubiquitous as a material for the synthesis of polycarbonate and epoxy resins [9], used in products ranging from small plastic water bottles and food packaging bags to large medical devices. However, as an endocrine disruptor, BPA can affect the nervous system, reproductive system, and immune system of humans and animals, and has estrogen-like and carcinogenic effects [10,11]. As substitutes for BPA, BPS and other substances have rapidly emerged to flood human life. Residues of BPS have been detected in the environment [12,13,14], and numerous reports have highlighted its potential hazards to human health, but there is a paucity of knowledge regarding its role in the dissemination of ARGs. Therefore, there is an urgent need to investigate the effect of BPS on the spread of antibiotic resistance.

The aim of this study was to investigate the impact of BPS on the propagation of ARGs and its possible mechanisms. A transformation system consisting of an exogenous DNA pUC19 plasmid and an engineered bacterium, *E. coli* DH5α, was established to test the influence of BPS at concentrations of 0.1–10 μg/mL. Furthermore, we investigated the activity of the oxidative stress system, alterations in cell membrane permeability and fluidity, and alterations in energy metabolism and flagellar motility. Additionally, we quantified the expression changes of genes associated with the increased transformation under BPS treatment using RT-qPCR. Our findings underscore the potential roles of BPS in the spread of antibiotic resistance through transformation.

## 2. Results

### 2.1. BPS Promotes the Spread of ARGs via Transformation

**Figure 1:**
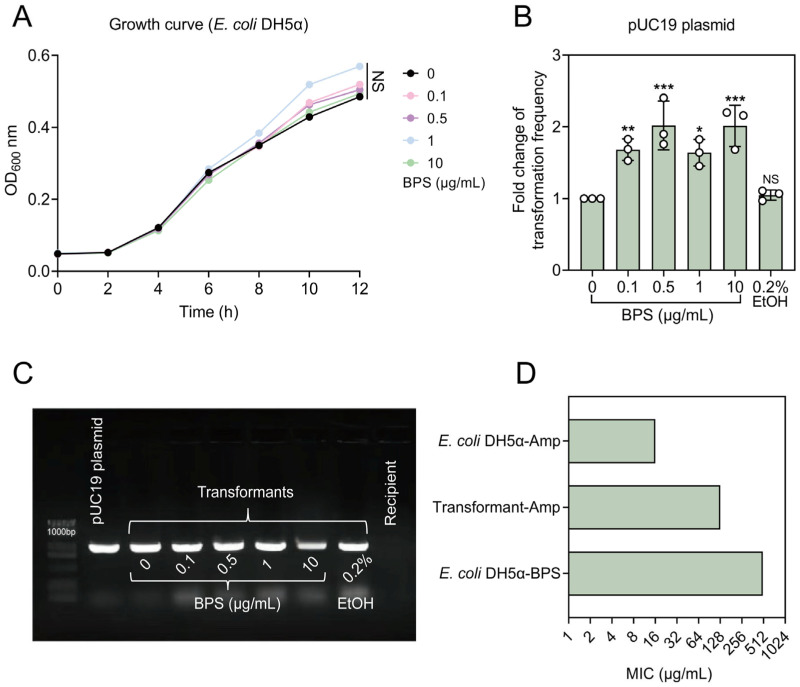
BPS promotes the transformation of ARGs into *E. coli* DH5α. (**A**) Growth curves of the recipient bacterium (*E. coli* DH5α) in the presence of different concentrations of the BPS (0.1–10 μg/mL). (**B**) Effects of different concentrations of the BPS on the frequency of transformation of pUC19 plasmid into *E. coli* DH5α. Statistically significant differences were determined using one-way ANOVA at * *p* < 0.05, ** *p* < 0.01 and *** *p* < 0.001, respectively. NS, not significant. (**C**) Gel electropherograms of pUC19 plasmid, recipient bacteria, and transformants at different concentrations of BPS. (**D**) MIC values of recipient bacteria and transformants.

First, we tested the effect of BPS at environmentally relevant concentrations (0.1–10 μg/mL) on the growth status of the recipient bacteria, and the results showed that all experimental concentrations of BPS had no effect on the normal growth of *E. coli* DH5α (Figure 1A). To assess whether BPS affects the uptake efficiency of exogenous DNA, we chose a common recombinant plasmid, pUC19, as the exogenous DNA and competent *E. coli* DH5α as the recipient. The transformation frequency was significantly increased by 1.64–2.02-fold upon stimulation with BPS at concentrations ranging from 0.1 to 10 μg/mL, with the most pronounced effect observed at a concentration of 0.5 μg/mL, resulting in a 2.02-fold increase. As BPS is soluble in ethanol, to eliminate the influence of solvents, we conducted the transformation in the presence of 0.2% ethanol as well, and the result indicated that ethanol had no impact on the transformation (Figure 1B). Two methods were employed to confirm the successful uptake of the pUC19 plasmid and to detect its presence and characteristics in the transformants. Firstly, the antibiotic susceptibility result showed that the MICs of all transformants for AMP were 128 times higher than that of the recipient, indicating the acquisition of the AMP resistance gene (Figure 1D). Next, plasmid extraction and PCR amplification of the AMP resistance gene showed that the transformants had clear bands of similar size to the donor plasmid, whereas the recipient bacteria did not (Figure 1C). On the contrary, the recipient bacteria did not have the AMP resistance gene, while also showing sensitivity to AMP, and these results all indicate that the antibiotic-resistant plasmid successfully entered the recipient bacteria. Together, our findings reveal that the addition of BPS significantly promotes the uptake of ARGs by *E. coli* DH5α.

### 2.2. BPS Enhances Bacterial Oxidative Stress and Activates SOS Response

**Figure 2:**
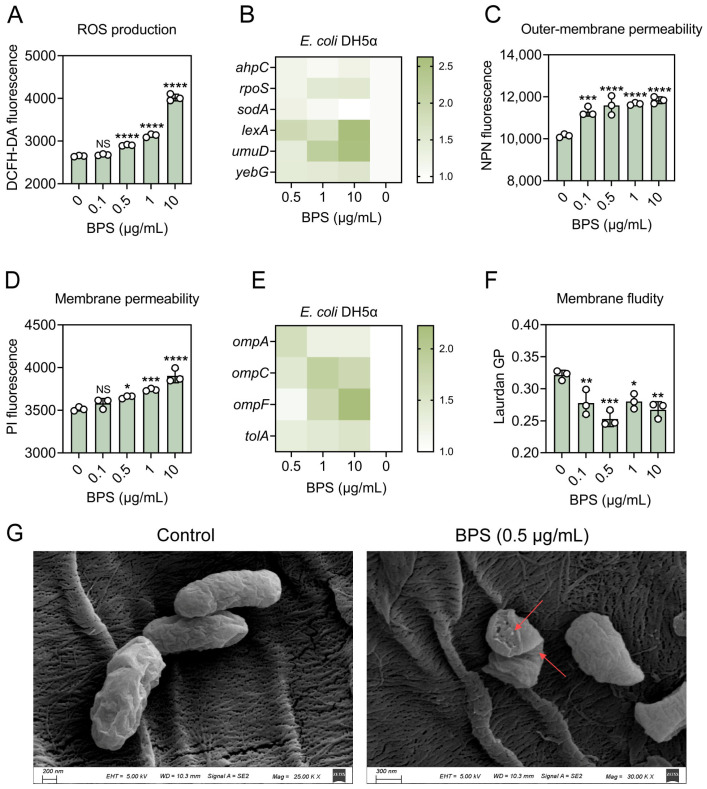
BPS stimulates the production of ROS and enhances membrane permeability in the recipient bacteria. (**A**) Effects of different concentrations of BPS on ROS production by recipient bacteria. (**B**) Heat map of increased expression levels of genes related to the oxidative stress system and SOS response system of bacteria after BPS treatment. (**C**) Changes in outer membrane permeability of recipient bacteria following BPS pressure. (**D**) Changes in inner membrane permeability in response to BPS treatment. (**E**) Effect of BPS on membrane fluidity. Statistically significant differences were determined using one-way ANOVA at * *p* < 0.05, ** *p* < 0.01, *** *p* < 0.001 and **** *p* < 0.0001, respectively. NS, not significant. (**F**) Heatmap of the increased expression levels of genes related to bacterial membrane permeability after BPS treatment. (**G**) SEM images of *E. coli* DH5α bacterial cells exposed to 0.5 μg/mL BPS for 4 h. Cell membrane damage is indicated by red arrows.

Next, we sought to elucidate the potential mechanisms by which BPS enhances the transformation of ARGs. ROS are important oxidative stress mediators, and it has been found that many factors can induce the overproduction of ROS to promote the transfer of ARGs. Given this, we hypothesized that BPS might stimulate ROS production and thus promote transformation, so we first assessed changes in ROS production in recipient bacteria after BPS treatment. ROS production was increased in all cases compared with the control, with 10 μg/mL BPS having the most pronounced effect, inducing a nearly 2.00-fold increase in ROS production (Figure 2A).

To confirm the changes in oxidative stress levels, we also monitored the expression levels of genes related to the oxidative stress system, such as *ahpC*, *rpoS,* and *sodA*. After exposure to 0.5–10 μg/mL BPS, the expression of *ahpC* and *rpoS* genes were significantly upregulated in bacteria, 1.10–1.26-fold, and 1.21–1.52-fold, respectively. As a subunit of the alkyl hydroperoxide reductase (AhpR) system, *ahpC* directly converts peroxidized substrates and thus protects the cells from suffering oxidative stress. As well, *rpoS* can help bacteria by regulating antioxidant defense against oxidative stress and enhance the possibility of bacterial DNA recombination, which ultimately promotes horizontal gene transfer. In addition, the expression of the *sodA* gene encoding superoxide dismutase was slightly upregulated (Figure 2B).

Excess ROS induce the SOS response, which is initiated when DNA is damaged, and it is claimed that SOS is also associated with HGT. In addition to stimulating ROS production, there is evidence that exposure to BPS also triggers the SOS response in recipient bacteria. Expression of the *umuD* gene, which encodes the DNA polymerase V protein, was stimulated over a variable range of 1.39–2.58-fold upregulation at all BPS dose levels. In addition, the expression of *yebG*, which is involved in the SOS response, was upregulated about 1.50-fold during BPS exposure. In contrast, *lexA*, a transcriptional inhibitor of the SOS response, could respond to DNA damage, and its expression was significantly upregulated by 2.62-fold at a BPS exposure concentration of 10 μg/mL (Figure 2B). These results indicate that BPS promotes transformation by stimulating ROS production and activating SOS response.

### 2.3. BPS Improves Membrane Permeability and Fluidity

The cell membrane of bacteria not only separates the internal and external environments of bacteria, but also allows information transfer and material exchange with the external environment, for example, by regulating the transfer of DNA and material exchange between inside and outside the cell to influence the efficiency of horizontal gene transfer. It has been shown that the overproduction of ROS increases the permeability of the cell membrane, and the increase in membrane permeability will be more favorable for the entry of plasmids. Cells in the sensory state have higher cell membrane permeability and porosity than non-sensory cells, which may facilitate DNA uptake by crossing the extracellular membrane barrier. Thus, we tested cell membrane permeability changes in the presence and absence of BPS. Consequently, a 1.11–1.17-fold rise in outer membrane permeability following treatment with 0.1–10 μg/mL of BPS was observed (Figure 2C). Similarly, we also found that the inner membrane permeability also showed a concentration-dependent enhancement (Figure 2D), reaching 1.11-fold at a concentration stimulation of 10 μg/mL BPS, and these results matched by ROS production.

As a class of typical membrane channel proteins in the family of outer membrane proteins (OMPs), *ompA* plays an important role in cell invasion, adhesion, and maintenance of the integrity of the outer membrane of the bacteriophage, whereas *ompC* allows passive diffusion of small molecules. The gene *tolA*, as one of the components of the Tol-Pal system (a composite protein system of the extracellular membrane), is important for both the maintenance of membrane permeability and the transport of substances. Therefore, we determined the relative expression of membrane permeability-related genes *ompA*, *ompC*, *ompF*, and *tolA*. We observed that the relative expression of *ompA*, *ompC*, *ompF*, and *tolA* genes increased 1.22–1.56-fold, 1.46–1.85-fold, 1.12–2.25-fold, and 1.30–1.58-fold, respectively, after bacteria were exposed to different concentrations of BPS (Figure 2E).

The transport of materials and the transduction of information in the cell are related to membrane fluidity, and it has been shown that the high fluidity of the cell membrane facilitates the entry and integration of exogenous DNA [15,16]. Bacterial cell membrane fluidity was detected using a Laurdan fluorescent probe. A significant decrease in GP values was found in all cases treated with BPS, down to 0.78-fold at a concentration of 0.5 μg/mL, which reflects the enhancement of membrane fluidity (Figure 2F).

Furthermore, in an attempt to get a more intuitive impression of the membrane damage caused by BPS, we observed the surface morphology of the recipient bacterium *E. coli* DH5α before and after 0.5 μg/mL BPS treatment with the help of SEM. The cell membrane structure of the control group was intact, and the surface was smooth and clear. In contrast, the cell membrane structure was disrupted in the treatment group with concomitant bacterial wrinkling and folding (Figure 2G). These results indicate that BPS can induce the enhancement of cell membrane permeability and fluidity and promote the expression of membrane protein-related genes, which contribute to the transformation process.

### 2.4. BPS Enhances Metabolic States by Accelerating TCA Cycle

**Figure 3:**
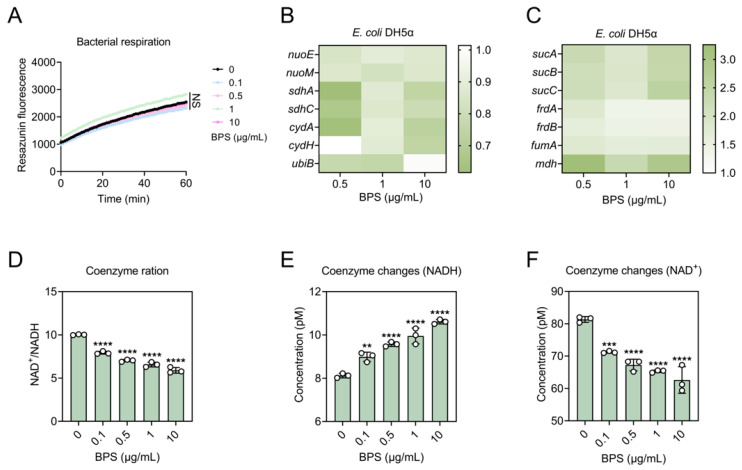
BPS enhances bacterial metabolism by accelerating the TCA cycle. (**A**) Bacterial respiration levels of *E. coli* DH5α were unchanged or even decreased under the pressure of BPS. (**B**) Heatmap of the expression levels of genes related to bacterial electron transport chain in response to BPS treatment. (**C**) Heatmap of TCA cycle-related gene expression levels in response to BPS. Bacterial (**D**) NAD^+^/NADH ratio, (**E**) NAD^+^ content, and (**F**) NADH content under BPS treatment. Statistically significant differences were determined using one-way ANOVA at ** *p* < 0.01, *** *p* < 0.001 and **** *p* < 0.0001, respectively. NS, not significant.

The accumulation of ROS represents a state of stress for the bacteria. Thus, we speculated that bacteria may fine-tune their respiratory and metabolic responses in response to this condition. To test this, we first examined the bacterial respiration level of the bacteria host using the resazurin. Surprisingly, the fluorescence intensity hardly changed after exposed to BPS compared to the control, indicating no significant change in the bacterial respiration level (Figure 3A). Meanwhile, regarding the effect of BPS on bacterial respiration levels, we used RT-qPCR to probe changes in the expression levels of complexes on the electron transport chain at the molecular level. The genes *nuoE* and *nuoM* encode important subunits on the NADH dehydrogenase module and the proton pump module, respectively, of complex I. The genes *sdhA* and *sdhC*, as members of the SDH complex, are important components of complex II. The genes *cydA* and *cydH*, subunits of cytochrome oxidase of complex IV, are involved in the catabolism of NADH and provide the impetus for the transmembrane movement of protons for ATP synthesis. The *ubiB* gene encodes an enzyme involved in the process of coenzyme Q synthesis. The relative expression levels of *nuoE*, *nuoM*, *sdhA*, *sdhC*, *cydA*, *cydH*, and *ubiB* genes were all downregulated after bacterial exposure to BPS, mostly by about 0.70- and 0.80-fold, with *sdhA*, *sdhC*, and *cydA* downregulated by about 0.60-fold when exposed to BPS with a concentration of 0.5 μg/mL (Figure 3B).

One of the most important functions of the tricarboxylic acid cycle (TCA) is to support the electron transport activities of protein complexes embedded in the inner mitochondrial membrane. To explore the impact of BPS stress on the metabolism of recipient bacteria, we conducted a typical gene expression analysis focusing on the TCA cycle. The genes *sucA*, *sucB*, *sucC* encoding 2-oxoglutarate dehydrogenase, dihydrothiooctanoyltranssuccinimidylase, succinyl coenzyme A synthase subunit β were upregulated 1.83–2.58-fold in response to BPS treatment. The relative expression levels of *frdA*, *frdB*, and *fumA* encoding fumarate reductase flavoprotein subunit, fumarate reductase iron-sulfur protein, and fumarate hydratase were upregulated 1.37–1.81-fold in response to the stress of BPS. *mdh*, a gene encoding malate dehydrogenase, was upregulated 2.35–3.24-fold in its expression level under the stress of BPS, and the most pronounced increase was detected when exposed to a concentration of 0.5 μg/mL BPS (Figure 3C). The TCA cycle is also a key biological process for generating reducing power, which produces most of the NADH required by the organism. As a key intermediate, an increase in NADH concentration can somehow indicate the activation of the TCA cycle. Therefore, we co-incubated BPS with bacteria and measured the intracellular NADH concentration of the bacteria. Consistently, we observed a distinct increase in NADH content accompanied by a decrease in NAD^+^/NADH, indicating an activation of the metabolic state (Figure 3D–F). These results suggest that BPS ultimately activates the metabolic state of the bacteria by accelerating the TCA cycle.

### 2.5. BPS Stimulates ATP Supply and Improves Flagellar Motility

**Figure 4:**
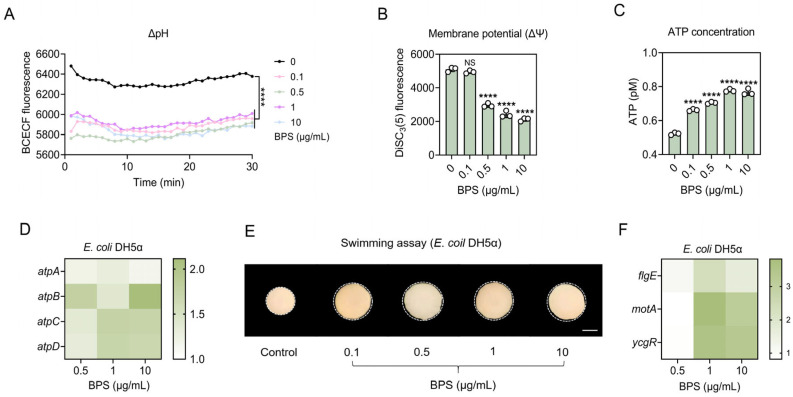
BPS stimulates ATP synthesis and flagellar motility. (**A**) ΔpH changes of recipient bacteria in response to BPS treatment, measured using BCECF. (**B**) Membrane potential of recipient bacteria in response to BPS stress, monitored using DiSC_3_(5). (**C**) Bacterial ATP synthesis after exposure to BPS. (**D**) Heat map of the expression level of bacterial ATP synthase-related genes under BPS stress. (**E**) Heatmap of the expression level of bacterial flagellum-related genes after BPS treatment. (**F**) Swimming motility test of *E. coli* DH5α under BPS stress, scale bar, 0.5 cm. Statistically significant differences were determined using one-way ANOVA at **** *p* < 0.0001. NS, not significant.

Central carbon metabolism maintains PMF by reducing NADH to provide protons, which consist of ΔpH and ΔΨ compensating for each other. The results showed that under the stimulation of BPS, the BCECF-AM fluorescence intensity was significantly decreased in the experimental group compared with control (Figure 4A), and the DiSC_3_(5) fluorescence intensity was significantly decreased when the bacteria were treated with high concentrations of BPS, up to 1.60–2.50-fold (Figure 4B). This indicated that intracellular ΔpH was broken and intracellular H^+^ increased; ΔΨ was enhanced and the transmembrane potential increased, both of which were in opposite trends, maintaining the relative stability of the PMF. The PMF plays an indispensable role in many biological activities, such as ATP synthesis, substrate transport, and flagellar motility [17].

When the ETC pumps electrons across the membrane, creating a potential difference, ATP is synthesized by the enzyme ATP synthase to fuel normal activities. As a substrate for the ETC, NADH is also affected by BPS, which may affect intracellular ATP generation. Based on this, we examined ATP levels, and the results showed that ATP levels were increased in a concentration-dependent manner when bacteria were exposed to tested concentrations of BPS (Figure 4C), reaching 1.49-fold at a concentration of 1 μg/mL. The expression levels of *atpA*, *atpB*, *atpC*, and *atpD*, which are important components of ATP synthase, were increased by 1.15–1.29-fold, 1.39–2.11-fold, 1.36–1.75-fold, and 1.31–1.71-fold, respectively, in the presence of BPS (Figure 4D). These results suggest that BPS drives ATP synthesis by accelerating the TCA cycle and thus provides an adequate energy source for plasmid integration.

The bacterial flagellum is an important organelle that runs across the bacterial surface, can be involved in motility and sensing, and can serve as a carrier involved in the transport of substances (facilitating the entry of exogenous plasmids into the cell). As an energy currency, the sufficiency of ATP facilitates more extensive and efficient uptake of plasmids by the flagellum. Therefore, we explored the level of flagellar motility using the swimming experiment. The results showed that the swimming radius of bacteria exposed to BPS on agar all increased compared to the control group (Figure 4E). We further explored bacterial motility by analyzing the expression of flagellum-related genes. The gene *flgE*, encoding the hook protein of the flagellum, which plays a crucial role in flagellar biosynthesis, swimming ability, and biofilm formation [18], was upregulated 1.15–2.35-fold in response to the pressure of BPS. The expression of *motA* and *ycgR* genes encoding flagellar motor proteins and flagellar braking proteins were upregulated 3.10–3.82-fold and 3.32–3.58-fold, respectively, in response to BPS (Figure 4F). These results demonstrate that BPS could induce the enhancement of flagellar motility by promoting ATP supply.

## 3. Discussion

The massive use of antibiotics gives rise to the evolutionary selection of antibiotic-resistant bacteria (ARBs), posing a disturbing threat to global public health. Gradually, the residual antibiotics in the environment were found to promote the spread of ARGs [19]. Even more frighteningly, it has recently been found that some non-antibiotic compounds play an important role in the dissemination of ARBs [8]. The contamination of bisphenol compounds is not only a factor on the environmental level, but also on the microbial level. It has been reported that BPA can upregulate pheromone expression, promote bacterial aggregation, and even act as a pheromone to directly activate conjugation [20]. In recent years, the use of BPS, which is the main substitute for BPA, has risen dramatically, and it has been used as a raw material for many daily products in almost all environments where humans have lived [21]; as their residues can be harmful to the environment and human beings, it is worthwhile to think about whether they have any effect on the spread of antibiotic resistance as well.

In this study, the common contaminant BPS was found to significantly promote the natural rate of transformation of plasmids carrying ARGs into *E. coli* DH5α over a range of relevant environmental concentrations (0.1 to 10 μg/mL) [22]. Our study also sought to determine the underlying mechanisms of how BPS promotes extracellular ARGs’ transformation from different aspects. Oxidative stress, one of the killing mechanisms of antimicrobial agents, is closely related to the development of antimicrobial resistance [23]. Some antimicrobials produce excess ROS within bacteria [24], which can (1) cause oxidative DNA damage, which may lead to genetic mutations and subsequent conversion of bacteria to antimicrobial resistance; (2) cause oxidative cell membrane damage that favors the uptake of extracellular components (such as ARGs); and (3) directly modulate the ability of human pathogens to lead to enhanced natural conversion. Thus, ROS play an important role in the generation and propagation of antibiotic resistance [23,25,26]. In previous studies, chemical-induced oxidative stress has also been suggested to promote the transformation of ARGs [27]. In this transformation study, we found that the level of ROS production increased significantly with increasing BPS concentrations, and that the increased level of ROS production in the recipient strains was a key factor in the accelerated transformation by BPS. Consistently, the high expression of antioxidant enzymes such as *sodA* belongs to the defense response to oxidative stress and helps bacteria to be exempted from suffering from excess ROS. In addition, the expression levels of DNA integration/repair genes such as *umuD*, *lexA*, and *yebG* were also increased, and the upregulation of the expression of these genes reflected the activation of the SOS response [28,29]. Among them, *umuD* is involved in bacterial mismatch repair, which may increase the chances of integration of exogenous DNA into the bacterial chromosome, leading to an increase in natural transformation [30].

The cell membrane serves as a barrier for free plasmids to enter the recipient bacteria [31], and previous studies have found that increased ROS production may lead to increased cell membrane permeability, resulting in impaired membrane barrier and enhanced plasmid transfer [7,32]. Our results indicated that the permeability of the inner and outer membranes of the recipient bacteria was significantly increased by the stimulation of BPS. In addition, SEM more visually demonstrated that BPS compromised the integrity of the membrane and caused bacterial shrinking, providing more pores for plasmid uptake, which is consistent with previous findings that exposure to colistin can lead to outer membrane damage, alter the normal membrane composition of *Acinetobacter baumannii*, and promote free DNA uptake [33].

Plasmid uptake requires sufficient energy, and an adequate energy source helps bacteria to undergo transformation. Interestingly, there was an increase in NADH and a decrease in NAD^+^ in the recipient bacteria exposed to BPS, which was a sign of an increased TCA cycle. Seven genes involved in the TCA cycle, *sucA*, *sucB*, *sucC*, *frdA*, *frdB*, *fumA*, and *mdh* were upregulated in the presence of BPS. Among them, *mdh* and *sucA* encode malate dehydrogenase and α-ketoglutarate dehydrogenase, respectively, which are involved in NADH production during the TCA cycle. Enzymes encoded by *frdB* are involved in the ETC and are important for the maintenance of membrane potential.

Notable upregulation of genes associated with the TCA cycle suggests that BPS compensates for perturbations in their energy metabolism by increasing TCA cycle activity to maintain their membrane potential and ATP levels [34]. Due to the impaired function of the complex, NADH could not be decomposed in time and sludged in the bacteria, which explains why intracellular H^+^, ATP, and NADH were elevated at the same time. On the other hand, the increased motility of the bacterial flagellum facilitates the promotion of the uptake of extracellular plasmids, a process that relies on a high level of bacterial energy metabolism [35]. Increased ATP synthesis provides energy and favorable conditions for plasmid internal translocation [36] and integration [37].

**Figure 5:**
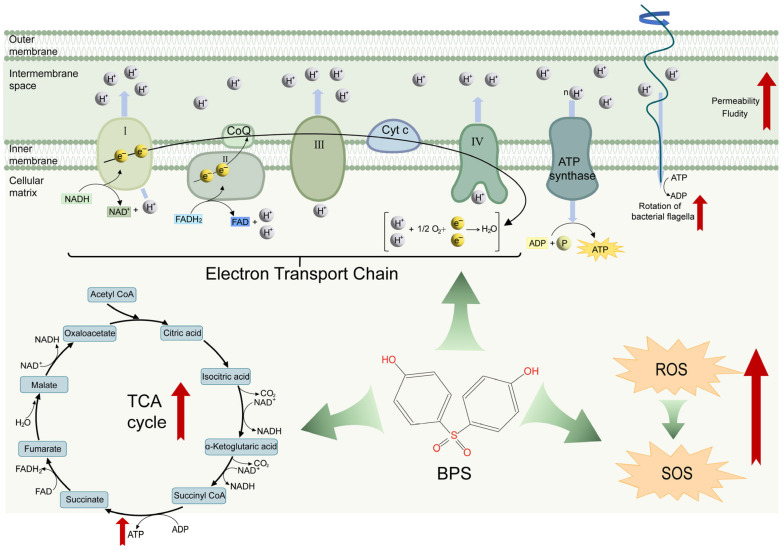
Schematic diagram of the mechanism of increased transformation by BPS treatment. The frequency of transformation of antibiotic-resistant plasmids was significantly increased under the stress of low concentrations of BPS. Potential mechanisms include a dramatic increase in ROS production and activation of the SOS response, which increases membrane permeability and fluidity. In addition, the accelerated TCA cycle generates a large amount of ATP, and flagellar motility was also enhanced. These actions are favorable for plasmid uptake, facilitation, and integration.

In conclusion, we demonstrate that BPS significantly promotes the transformation of exogenous ARGs. BPS exposure leads to an increase in membrane permeability by stimulating the production of ROS in bacteria, which activates the SOS response. Furthermore, BPS-induced activation of the TCA cycle promotes ATP production, which facilitates flagellar formation and bacterial motility (Figure 5). Considering the role of bisphenol compounds in promoting the spread of antibiotic resistance, their contamination in the environment needs continuous monitoring.

## 4. Materials and Methods

### 4.1. Bacterial Strains, Plasmids, Antibiotics, BPS, and Culture Media

*E. coli* DH5α is preserved in our laboratory. The pUC19 plasmid is a common cloning plasmid vector with the ampicillin (AMP) resistance gene. The bisphenol compound BPS was purchased from Yuan Ye Biotechnology (Shanghai, China), ampicillin sodium was purchased from the China Veterinary Control Institute, and LB nutrient agar was purchased from Qingdao Hope Biotechnology (Qingdao, China).

### 4.2. Measurement of Growth Curves

An *E. coli* DH5α single colony was picked for overnight incubation and then diluted into appropriate LB broth at 1:1000. Subsequently, the final concentrations of 0, 0.1, 0.5, 1, 5, and 10 μg/mL of BPS were added and placed in a 37 °C constant-temperature incubator. Fluorescence intensity at 600 nm was measured at hourly intervals for 12 h using an Infinite E Plex enzyme labeler (Tecan). Three biological replicates were set up for all experiments.

### 4.3. Determination of Minimum Inhibitory Concentration (MIC)

The MIC values of the recipient bacteria and transformants were tested according to the European Committee for Antimicrobial Susceptibility Testing (EUCAST) guidelines. Bacteria were cultured overnight and diluted 1000-fold in 96-well petri dishes containing MH broth and different concentrations of the BPS to approximately 10^5^ CFU/mL. The MIC value was determined as the concentration of antibiotic that inhibited the growth of 90% of the bacteria after 18 h.

### 4.4. Establishment of Transformation System and Validation of Transformants

*E. coli* DH5α was used as the recipient bacterium and pUC19 plasmid as the exogenous DNA to establish the transformation model. Specifically, the pUC19 plasmid was added to pre-cooled competent cell, followed by the addition of BPS with final concentrations of 0, 0.01, 0.1, 0.5, 1, 10 μg/mL, incubated on ice for 30 min and then heat-excited at 42 °C for 45 s, and then immediately ice-bathed for 2–3 min, and each tube of the sample was added with 900 μL of LB broth and incubated in a shaker at 37 °C for 1 h. Subsequently, the samples were plated onto antibiotic-free LB agar petri dishes as well as petri dishes containing 100 mg/L AMP LB agar, and then incubated at 37 °C for 12–16 h. The transformants were screened and the transformation frequency (TF) was calculated: TF = *N_T_/N_R_*, where *N_T_* stands for the number of transformants and *N_R_* stands for the number of recipients. PCR analysis and gel electrophoresis were performed to identify the accuracy of transformants. The reaction conditions were set as follows: pre-denaturation at 95 °C for 10 min, denaturation at 95 °C for 30 s, annealing at 55 °C for 30 s, extension at 72 °C for 1 min, and cycling for 30 times.

### 4.5. Detection of Oxidative Stress Levels in Bacteria

Oxidative stress levels were determined using a cellular ROS assay kit (Beyotime, Shanghai, China). Briefly, *E. coli* DH5α was cultured overnight and washed with 0.9% NaCl to an OD_600_ of 0.5. Then, 2′,7′-dichlorodihydrofluorescein diacetate (DCFH-DA) was added to the bacterial suspension at a final concentration of 10 μM, and then the suspension was incubated at 37 °C for 30 min at 200 r/min in the dark, and then washed with 0.9% NaCl twice to remove the unbound probes. The addition of different concentrations of BPS acted for 1 h. Then 200 μL of each well was spread onto a 96-well black flat-bottomed plate, and the fluorescence intensity was measured with an excitation wavelength of 488 nm and emission wavelength of 525 nm.

### 4.6. Detection of Bacterial Membrane Permeability and Fluidity

*E. coli* DH5 was cultured to an OD_600_ of 0.5. For the bacterial outer membrane permeability assay, a final concentration of 10 μM *N*-phenyl-1-naphthylamine (NPN, Shanghai, China) was added to the bacterial suspension and incubated at 37 °C, 200 r/min for 30 min. Subsequently, different concentrations of BPS were added to act for 1 h. Then, the fluorescence intensity was measured with an excitation wavelength at 350 nm and an emission wavelength at 420 nm. When detecting the permeability of the bacterial inner membrane, a final concentration of 10 μM propylenediamine iodide (PI, Beyotime, Shanghai, China) was used, and the rest of the operation was the same as that described above with an excitation wavelength of 535 nm and emission wavelength of 615 nm. Additionally, 10 μM of Laurdan was used for the measurement of membrane mobility, with an excitation wavelength of 350 nm and emission wavelengths of 435 and 490 nm, and the Laurdan GP was calculated as GP = (I_435_ − I_490_)/(I_435_ + I_490_).

### 4.7. Scanning Electron Microscope (SEM) Analysis

*E. coli* DH5α was pretreated following the aforementioned method, and 0.5 μg/mL BPS was added to the bacterial suspension and incubated at 37 °C for 4 h. Subsequently, centrifugation was carried out, the suspension was washed three times with 0.9% NaCl, the supernatant was discarded, and 2.5% glutaraldehyde was added in the ratio of 1:10, mixed, and fixed at 4 °C overnight. Gradient dehydration was then performed as directed, and the samples were then dried, tacked, gold-plated, and visualized on a Gemini SEM 300.

### 4.8. Detection of Bacterial NAD^+^/NADH

A NAD^+^/NADH assay kit (WST-8) was used for the determination of NAD^+^/NADH. An *E. coli* DH5α culture with an optical density of 0.5 at OD_600_ was treated with different concentrations of BPS for 4 h, washed with 0.9% NaCl twice, centrifuged, and the supernatant was discarded; then, it was added with 200 μL of pre-cooled NAD^+^/NADH extract and mixed. The supernatant was centrifuged and divided into two parts, and one part of the supernatant was incubated with the working solution for 10 min, then incubated with the color-developing solution for 30 min, and the OD_450_ was measured to get the total amount of NAD^+^ and NADH. The other part was subjected to a PCR reaction at 60 °C for 30 min, and the subsequent operation was the same as the above operation to obtain the amount of NADH.

### 4.9. Detection of Bacterial Proton Motive Force (PMF)

ΔpH was determined using a BCECF-AM fluorescent probe at a final concentration of 10 μM. The bacteria were subjected to pretreatment using the aforementioned method, added to the probe, and incubated for 30 min away from light, after which BPS was added and measured at 1 min intervals for one hour, with an excitation wavelength of 488 nm and emission wavelength of 535 nm. The ΔΨ was determined using a DiSC_3_(5) probe at a final concentration of 0.5 μM. The bacterial suspension was centrifuged, resuspended in 0.9% NaCl, and incubated with the probe for 30 min in the dark, and then incubated with BPS for 1 h. The fluorescence intensity was measured at an excitation wavelength of 622 nm and an emission wavelength of 670 nm.

### 4.10. Assay of Bacterial Motility

LB broth containing 0.3% (*w*/*v*) agar powder was configured and mixed with different concentrations of BPS before it solidified. Then, 2 μL of bacteria were adhered to each medium right in the center, and the bacteria in the medium were placed to incubate at 37 °C for at least 48 h. The inhibition bands were then photographed, and the diameter of the bacteria was measured.

### 4.11. Monitoring of Bacterial Respiration Levels

The respiration level was determined using resazurin [38] at a final concentration of 0.1 μg/mL. The bacteria with an OD_600_ of 0.5 were introduced to the resazurin and incubated in a light-free environment for 30 min, and then directly measured for fluorescence intensity for 1 h at an excitation wavelength of 550 nm and an emission wavelength of 590 nm after adding the BPS.

### 4.12. Content Determination of Bacterial ATP

ATP levels were determined using an ATP assay kit (Beyotime, Shanghai, China). *E. coli* DH5α suspension was resuspended with NaCl until an OD_600_ of 0.5, and different concentrations of BPS were added and acted for 1 h. According to the instruction, each sample was centrifuged, washed with 0.9% NaCl, and pre-cooled ATP test reagent was added sequentially, and the supernatant of the centrifuged solution was taken to measure the intracellular ATP level.

### 4.13. RNA Extraction and RT-qPCR Analysis

Overnight *E. coli* DH5α was cultured in fresh broth until logarithmic phase, centrifuged, resuspended in 0.9% NaCl until OD_600_ of 0.5, and incubated with different concentrations of BPS for 4 h. Subsequently, RNA was extracted with the RNA-easy Isolation Reagent (Vazyme Biotech, Nanjing, China). The extracted RNA was reverse transcribed into cDNA using the HiScript^®^ III RT Super Mix for qPCR (+gDNA wiper) Kits (Vazyme). RT-PCR was performed using the ChamQ^TM^ SYBR^®^ Color qPCR Master Mix Kits (Vazyme Biotechnology, China), which was paired with optimized primers, with 16s as the internal reference gene, and the 2^−ΔΔCt^ method was adopted as the relative expression of membrane permeability-, ATP level-, flagellar synthesis-, and secretion system-related genes after exposure to BPS. The reaction process was divided into four steps: pre-denaturation at 95 °C for 30 s, denaturation at 95 °C for 10 s, primer annealing at 60 °C for 30 s, extension at 72 °C for 30 s, and cycling for 40 times. The analysis was performed using a 7500 fast real-time PCR system (Applied Biosystems, Foster City, CA, USA). The primer sequences are listed in Table S1. Significance analysis of RT-qPCR is provided in Table S2.

### 4.14. Statistical Analysis

All data were analyzed using GraphPad version 9.5 software. All data are expressed as mean ± SD and *p* values were calculated using one-way analysis of variance (ANOVA) between multiple groups. Significance levels are indicated by asterisks: * *p* < 0.05, ** *p* < 0.01, *** *p* < 0.001 and **** *p* < 0.0001. NS was not significant.

## Data Availability

Data are contained within the article.

## Supplementary Materials

The following supporting information can be downloaded at: https://www.mdpi.com/article/10.3390/ijms25189819/s1.

## References

[1] Darby E.M., Trampari E., Siasat P., Gaya M.S., Alav I., Webber M.A., Blair J.M.A. (2022). Molecular mechanisms of antibiotic resistance revisited. Nat. Rev. Microbiol..

[2] Bhardwaj S., Mehra P., Dhanjal D.S., Sharma P., Sharma V., Singh R., Nepovimova E., Chopra C., Kuca K. (2022). Antibiotics and Antibiotic Resistance-Flipsides of the Same Coin. Curr. Pharm. Des..

[3] Kohanski M.A., DePristo M.A., Collins J.J. (2010). Sublethal antibiotic treatment leads to multidrug resistance via radical-induced mutagenesis. Mol. Cell.

[4] Lopatkin A.J., Huang S., Smith R.P., Srimani J.K., Sysoeva T.A., Bewick S., Karig D.K., You L. (2016). Antibiotics as a selective driver for conjugation dynamics. Nat. Microbiol..

[5] Yu Z., Wang Y., Henderson I.R., Guo J. (2022). Artificial sweeteners stimulate horizontal transfer of extracellular antibiotic resistance genes through natural transformation. ISME J..

[6] Liao J., Chen Y., Huang H. (2019). Effects of CO_2_ on the transformation of antibiotic resistance genes via increasing cell membrane channels. Environ. Pollut..

[7] Zhu S., Yang B., Jia Y., Yu F., Wang Z., Liu Y. (2023). Comprehensive analysis of disinfectants on the horizontal transfer of antibiotic resistance genes. J. Hazard. Mater..

[8] Weatherly L.M., Gosse J.A. (2017). Triclosan exposure, transformation, and human health effects. J. Toxicol. Environ. Health B Crit. Rev..

[9] Ma Y., Liu H., Wu J., Yuan L., Wang Y., Du X., Wang R., Marwa P.W., Petlulu P., Chen X. (2019). The adverse health effects of bisphenol A and related toxicity mechanisms. Environ. Res..

[10] Manzan-Martins C., Paulesu L. (2021). Impact of bisphenol A (BPA) on cells and tissues at the human materno-fetal interface. Tissue Cell.

[11] Santoro A., Chianese R., Troisi J., Richards S., Nori S.L., Fasano S., Guida M., Plunk E., Viggiano A., Pierantoni R. (2019). Neuro-toxic and Reproductive Effects of BPA. Curr. Neuropharmacol..

[12] Lao X., Tam N.F.Y., Zhong M., Wu Q., Liu Z., Huang X., Wei L., Liu Y., Luo D., Li S. (2024). Distribution and risk assessment of antibiotic and bisphenol compounds residues in drinking water sources of Guangdong. Environ. Earth Sci..

[13] Zhang J., Yao K., Yin J., Lyu B., Zhao Y., Li J., Shao B., Wu Y. (2022). Exposure to Bisphenolic Analogues in the Sixth Total Diet Study—China, 2016–2019. China CDC Wkly..

[14] Gingrich J., Pu Y., Roberts J., Karthikraj R., Kannan K., Ehrhardt R., Veiga-Lopez A. (2018). Gestational bisphenol S impairs placental endocrine function and the fusogenic trophoblast signaling pathway. Arch. Toxicol..

[15] Sreedevi P.R., Suresh K. (2023). Cold atmospheric plasma mediated cell membrane permeation and gene delivery-empirical interventions and pertinence. Adv. Colloid Interface Sci..

[16] Cao P., Wall D. (2020). The Fluidity of the Bacterial Outer Membrane Is Species Specific: Bacterial Lifestyles and the Emergence of a Fluid Outer Membrane. Bioessays.

[17] Le D., Krasnopeeva E., Sinjab F., Pilizota T., Kim M. (2021). Active Efflux Leads to Heterogeneous Dissipation of Proton Motive Force by Protonophores in Bacteria. mBio.

[18] You Y., Ye F., Mao W., Yang H., Lai J., Deng S. (2023). An overview of the structure and function of the flagellar hook FlgE protein. World J. Microbiol. Biotechnol..

[19] Arsene M.M.J., Davares A.K.L., Viktorovna P.I., Andreevna S.L., Sarra S., Khelifi I., Sergueievna D.M. (2022). The public health issue of antibiotic residues in food and feed: Causes, consequences, and potential solutions. Vet. World.

[20] Yang Y., Yang X., Zhou H., Niu Y., Li J., Fu X., Wang S., Xue B., Li C., Zhao C. (2022). Bisphenols Promote the Pheromone-Responsive Plasmid-Mediated Conjugative Transfer of Antibiotic Resistance Genes in Enterococcus faecalis. Environ. Sci. Technol..

[21] Wu L.H., Zhang X.M., Wang F., Gao C.J., Chen D., Palumbo J.R., Guo Y., Zeng E.Y. (2018). Occurrence of bisphenol S in the environment and implications for human exposure: A short review. Sci. Total Environ..

[22] Chen D., Kannan K., Tan H.L., Zheng Z.G., Feng Y.L., Wu Y., Widelka M. (2016). Bisphenol Analogues Other Than BPA: Environmental Occurrence, Human Exposure, and Toxicity-A Review. Environ. Sci. Technol..

[23] Zhang S., Wang Y., Lu J., Yu Z., Song H., Bond P.L., Guo J. (2021). Chlorine disinfection facilitates natural transformation through ROS-mediated oxidative stress. ISME J..

[24] Johnston C., Martin B., Fichant G., Polard P., Claverys J.P. (2014). Bacterial transformation: Distribution, shared mechanisms and divergent control. Nat. Rev. Microbiol..

[25] Ma L.N., Mi H.F., Xue Y.X., Wang D., Zhao X.L. (2016). The mechanism of ROS regulation of antibiotic resistance and antimicrobial lethality. Yi Chuan.

[26] Zhang S., Yang M.J., Peng B., Peng X.X., Li H. (2020). Reduced ROS-mediated antibiotic resistance and its reverting by glucose in Vibrio alginolyticus. Environ. Microbiol..

[27] Mantilla-Calderon D., Plewa M.J., Michoud G., Fodelianakis S., Daffonchio D., Hong P.Y. (2019). Water Disinfection Byproducts Increase Natural Transformation Rates of Environmental DNA in Acinetobacter baylyi ADP1. Environ. Sci. Technol..

[28] Zhang Y., Gu A.Z., He M., Li D., Chen J. (2017). Subinhibitory concentrations of disinfectants promote the horizontal transfer of multidrug resistance genes within and across genera. Environ. Sci. Technol..

[29] Yu Z., Wang Y., Lu J., Bond P.L., Guo J. (2021). Nonnutritive sweeteners can promote the dissemination of antibiotic resistance through conjugative gene transfer. ISME J..

[30] Simon S.M., Sousa F.J., Mohana-Borges R., Walker G.C. (2008). Regulation of Escherichia coli SOS mutagenesis by dimeric intrinsically disordered umuD gene products. Proc. Natl. Acad. Sci. USA.

[31] Kulbacka J., Choromanska A., Rossowska J., Wezgowiec J., Saczko J., Rols M.P. (2017). Cell Membrane Transport Mechanisms: Ion Channels and Electrical Properties of Cell Membranes. Adv. Anat. Embryol. Cell Biol..

[32] Lu J., Wang Y., Zhang S., Bond P., Yuan Z., Guo J. (2020). Triclosan at environmental concentrations can enhance the spread of extracellular antibiotic resistance genes through transformation. Sci. Total Environ..

[33] Henry R., Crane B., Powell D., Deveson Lucas D., Li Z., Aranda J., Harrison P., Nation R.L., Adler B., Harper M. (2015). The transcriptomic response of Acinetobacter baumannii to colistin and doripenem alone and in combination in an in vitro pharmacokinetics/pharmacodynamics model. J. Antimicrob. Chemother..

[34] Zhou Y., Yong Y., Zhu C., Yang H., Fang B. (2022). Exogenous D-ribose promotes gentamicin treatment of several drug-resistant Salmonella. Front. Microbiol..

[35] Judge A., Dodd M.S. (2020). Metabolism. Essays Biochem..

[36] Alvarez-Rodriguez I., Arana L., Ugarte-Uribe B., Gomez-Rubio E., Martin-Santamaria S., Garbisu C., Alkorta I. (2020). Type IV Coupling Proteins as Potential Targets to Control the Dissemination of Antibiotic Resistance. Front. Mol. Biosci..

[37] Martin I.V., MacNeill S.A. (2002). ATP-dependent DNA ligases. Genome Biol..

[38] Mehring A., Erdmann N., Walther J., Stiefelmaier J., Strieth D., Ulber R. (2021). A simple and low-cost resazurin assay for vitality assessment across species. J. Biotechnol..

