# Supplementary material for: Bisphenol S Promotes the Transfer of Antibiotic Resistance Genes via Transformation
Source: Int J Mol Sci. 2024 Sep 11;25(18):9819. doi: 10.3390/ijms25189819 (PMC11431945; doi:10.3390/ijms25189819)
Supplement: Supplementary file 1 [file ijms-25-09819-s001.zip › Supplementary Information.pdf]

Supplementary Information

Table S1. Primer sequences used in this study.

Genes	Sequences (5'-3')
ompA	F: TGAGCCTGGGTGTTTCCTA
	R: CAGAGCAGCCTGACCTTCC
ompC	F: AAGTAGTAGGTAGCACCAACATCA
	R: GGGCGAACAAAGCACAGAA
ompF	F: GGTCTGCGTCCGTCCAT
	R: GGTTGCGCCCACTTCA
tolA	F: ATGTCAGCAGCGACGGTAAA
	R: GCCAGACTTGGCGTTTCATC
ahpC	F: GGCAATCGAAGTTACCGCTG
	R: CAGAGACGGAGCCAGAGTTG
rpoS	F: TTTTACCACCAGACGCAAGT
	R: GGAACTGTTATCGCAGGGAG
nuoE	F: GTCGCCATGTGATCCGTTAT
	R: GTTTGCCCTGGTTTGATGTTC
nuoM	F: CTACCTTTGGGCTGGTCTTT
	R: GTTCCTGGCTGGCAATCT
sdhA	F: CCTGAACCGCTGGAACAATA
	R: CACGGAAGACCGAGAAGTTATG
sdhC	F: CGTAGGTATTCGCCACATGAT
	R: AGAAGTGAAAGCACGACAGTAA
cydA	F: CAGGAAGAGGAGACGAACAAAT
	R: AGCTCTTTCAGGCCGATAAC
cydH	F: ATGAGCACCGACCTTAAAT
	R: AGTCAAACCCACGGCTAC
uniB	F: GGTGTTCTCTACTCAGGTCTTTC
	R: TTCCGGGTGTTCATAGCTTAC
sucA	F: CAGTGCTGGCGTTTGAATATG
	G: GAGGAGATGAACTGGTCGATAAC
sucB	F: CGTGATGAAGTGCTGGTAGAA
	G: CAAGGATCTGACGAGACGTTAC
sucC	F: CTCCGCACCTGATCCATAAA
	G: GTTTACCTTCCAGACCCACTT
frdA	F: AGTACAGCGACGTGAAGATTAC
	G: CCATTCGCCTTCTCCTTCTT
frdB	F: GCTATAACCCGGAAGTCGATAC
	R: CGCATCCAGTAATGAGGTAGTT
fumA	F: TATCCCGTTTCTACACGCTTATC
	R: CCGTTATCCATCCGCTCTTT

Mdh	F: GAAGTTGAAGTGCCGGTTATTG
	G: CCACTTCCTGCTCGGTAAA
atpA	F: TTTGCATCCGACCTTGACGA
	R: CCAGGTAACCACGTTCTGCT
atpB	F: CGTACATTCTCGCTGGTGGA
	R: CGCCTTTTTGGCTACGCTAC
atpC	F: AGCACGGTCACGAAGAGTTT
	R: AGCCTTACGTTTCGCTTCCA
atpD	F: AGGGCGGTAAAGTTGGTCTG
	R: TGGAGTCGGTCATTTCGTGG
flgE	F: GACTATTCAGCAAGGGGCGA
	R: GCGTAACAGTAGGAAGCGGA
motA	F: ACGCTCTATTCCAGCGAACG
	R: TCCTCGGTTGTCGTCTGTTG
ycgR	F: GGCGCATTACTGGAAACAGC
	R: GAAAAACACCCCATTGCCCC
umuD	F: GCCCGACGGTACAGCTTATT
	R: ACACCAAAGACATCCAGCGT
lexA	F: GGTCGTTGTCGCACGTATTG
	R: TGAAGCTCTGCTGACGAAGG
yebG	F: CATTCGTGAGGGCGAAGAGA
	R: GAAAGGGCTTCACGTTGCTC
16S rRNA	F: CCTACGGGAGGCAGCAG
	R: ATTACCGCGGCTGCTGG

Table S2. Statistical analysis for the RT-qPCR expression levels of relevant genes.

Genes	BPS (μg/mL)		
	0.5	1	10
ompA	0.000233	0.00515	0.010215
ompC	0.001421	0.005154	0.017183
ompF	0.000712	0.003964	0.008979
tolA	0.000008	0.042634	0.037201
ahpC	0.001859	0.235666	0.026437
rpoS	0.027747	0.00429	0.000677
sodA	0.035214	0.46345	0.690247
lexA	0.005989	0.035712	0.017506
umuD	0.019682	0.105741	0.026323
yebG	0.13668	0.203725	0.012278
nuoE	0.120867	0.946151	0.784969
nuoM	0.222522	0.217052	0.008479
sdhA	0.0049	0.022113	0.592039
sdhC	0.000036	0.079772	0.402148
cydA	0.014732	0.056898	0.038905
cydH	0.49535	0.018555	0.150194
ubiB	0.116125	0.12608	0.711254
sucA	0.107448	0.02043	0.175765
sucB	0.000603	0.02523	0.00367
sucC	0.126185	0.073653	0.010583
frdA	0.004719	0.089612	0.034087
frdB	0.028405	0.09277	0.000684
fumA	0.004462	0.000243	0.063484
mdh	0.015963	0.000111	0.134474
atpA	0.000891	0.003843	0.44662
atpB	0.002508	0.127102	0.000459
atpC	0.010273	0.005948	0.000542
atpD	0.000487	0.00059	0.000368
flgE	0.254786	0.022904	0.170923
motA	0.000071	0.000008	0.000167
ycgR	0.047405	0.000052	0.000118
